# Examiners’ influence on the measured active and passive extension deficit in finger joints affected by Dupuytren disease

**DOI:** 10.1186/s12874-018-0577-8

**Published:** 2018-10-29

**Authors:** Jesper Nordenskjöld, Stina Brodén, Isam Atroshi

**Affiliations:** 1 Department of Orthopedics, Kristianstad-Ystad-Hässleholm Hospitals, Hässleholm, Sweden; 2 Department of Rehabilitation, Kristianstad Hospital, Kristianstad, Sweden; 3 Department of Clinical Sciences - Orthopedics, Lund University, Lund, Sweden

**Keywords:** Dupuytren disease, Hand surgery, Outcome measures

## Abstract

**Background:**

The most commonly reported outcome measure in Dupuytren disease is the extension deficit in finger joints. This study aimed to investigate the examiners’ influence on the measured difference between active and passive extension deficit.

**Methods:**

A prospective cohort study was conducted on 157 consecutive patients (81% men, mean age 70 years) scheduled for collagenase treatment for Dupuytren disease. Before injection, one of three experienced hand therapists measured active extension deficit (AED) and passive extension deficit (PED) in the metacarpophalangeal (MCP) and proximal interphalangeal (PIP) joints of the affected fingers using a hand-held metal goniometer. We included joints with ≥10° AED, and calculated mean AED and PED in MCP and PIP joints measured by each examiner. For adjusted analysis we used a mixed effects model to determine the relationship between the examiner and the AED-PED difference.

**Results:**

For all 291 joints measured, mean AED was 46° (SD 21) and mean PED was 37° (SD 23). Mean difference between AED and PED measured by examiner 1 was 6° (SD 6), by examiner 2 was 9° (SD 9), and by examiner 3 was 12° (SD 9). The mixed effects model analysis showed that the identity of the examining therapist was a significant determinant of the AED-PED difference.

**Conclusions:**

In Dupuytren disease measurement of active and passive extension deficit in finger joint contractures may vary significantly between different examiners. This must be taken into consideration when designing clinical studies and comparing outcomes between studies.

## Background

Joint range of motion (ROM) is one of the most important orthopedic outcome measures. For certain conditions, such as Dupuytren disease, a common fibroproliferative hand disorder [1–3], it is almost always considered as the primary outcome [4]. A large number of patients with Dupuytren disease develop finger joint contractures requiring treatment [5]. Surgical fasciectomy has traditionally been the most common treatment [5–7], but use of minimally invasive treatment methods such as collagenase injections [8, 9] and needle fasciotomy [10] has gained in popularity in recent years. A recent Cochrane review concluded that there is insufficient evidence to show superiority among different surgical treatment methods and highlighted difficulties in comparing studies due to inconsistent outcome measurement methodology and reporting [11].

The most important and commonly reported outcome measure of Dupuytren disease severity and treatment effect is ROM of affected finger joints [4], and more specifically the extension deficit. The measurement is usually performed using a goniometer, which is regarded as a reliable assessment tool [12]. In the literature, the extension deficit is reported as active extension deficit (AED) [13–15] and/or passive extension deficit (PED) [9, 10], although some studies do not specify how the extension deficit was measured [11, 12].

When different examiners measure joint ROM, examiner-related variability may affect measurement reliability [16, 17]. This may in turn affect the reported treatment results and subsequently comparison between studies. In this study of patients with Dupuytren disease we aimed to investigate the influence of the examiner on the size of difference between active and passive extension deficit in finger joint contractures measured by different examiners before treatment.

## Methods

### Participants

Between August 2014 and December 2015 a prospective cohort study was conducted at one orthopedic department (Kristianstad-Ystad-Hässleholm hospitals) in southern Sweden. The study participants were patients with Dupuytren disease scheduled for collagenase treatment. The indication for collagenase treatment was presence of a palpable cord and AED of ≥20° in the metacarpophalangeal (MCP) and/or proximal phalangeal (PIP) joint.

### Measurements

**Fig. 1:**
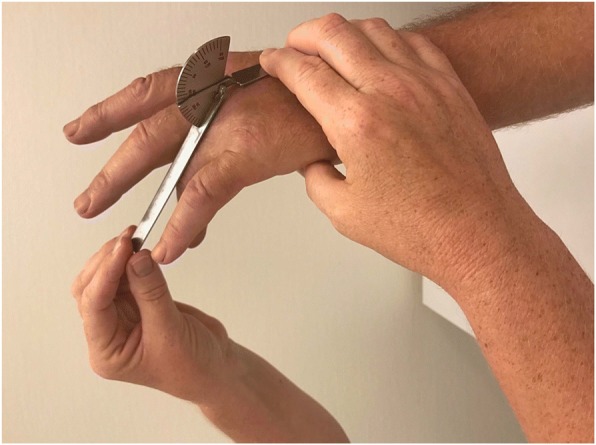
Measurement of MCP joint active extension deficit

**Fig. 2:**
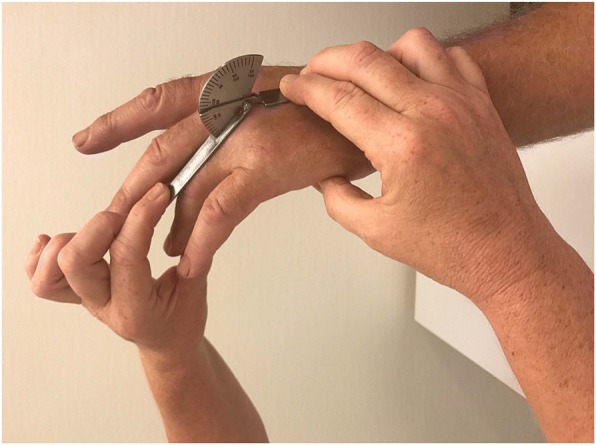
Measurement of MCP joint passive extension deficit

**Fig. 3:**
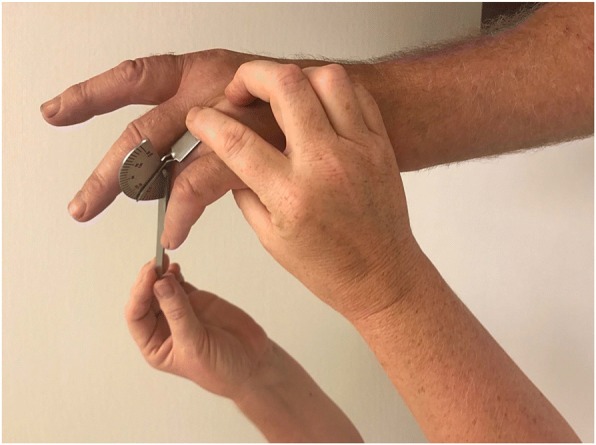
Measurement of PIP joint active extension deficit

**Fig. 4:**
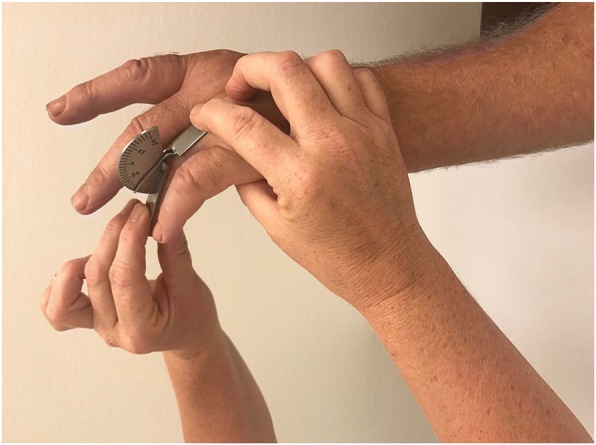
Measurement of PIP joint passive extension deficit

Immediately before collagenase injection the patients were examined by one of three examiners (experienced hand therapists). The assignment of therapists to the outpatient treatment sessions followed their work schedule independent of patient scheduling. The examining hand therapist used a standardized protocol [18] for measurements using a hand-held metal goniometer (Baseline® metal goniometer). The measurements included both AED and PED of the MCP and PIP joints of the affected fingers (Figs 1, 2, 3 and 4). During measurement the patient had the elbow in flexed position resting on the examination table and the forearm and wrist in neutral position. First, the examiner asked the patient to actively extend the fingers as much as possible and measured AED of each joint, with full extension and hyperextension recorded as 0° of extension deficit. Measurement of AED in the PIP joint was performed with the MCP joint actively extended, to standardize the phenomenon of dynamism [19]. After measuring AED, the examiner measured PED by applying pressure on the finger to extend the joints until resistance was felt. Measurement of PED in the PIP joint was performed with the MCP joint in maximum possible active extension.

### Statistical analysis

In a previous study investigating the inter-observer agreement in total PED in Dupuytren disease a mean difference of 2.1° (SD 10.3) in the left ring finger could be observed [17]. To be able to show a difference of at least 2.1° in PED between examiners with SD of 10.3, *p*-value of 0.05 and statistical power of 80%, we calculated a sample size of 189 joints. We included joints (MCP or PIP) in the treated fingers with ≥10° of AED. We calculated mean AED and PED for the MCP and PIP joints measured by each of the three examiners. We used the t-test to compare the AED-PED differences according to joint (MCP vs PIP), finger (small vs ring), and gender (men vs women) and analyzed the correlation with age using the Pearson correlation coefficient (r). We performed a mixed effects model analysis (patients and fingers as random effects) and a fixed effects model with robust variance (to account for patients providing multiple measurements) to determine the relationship between the size of the difference between AED and PED and the identity of the examiner, adjusting for affected joint, finger, gender, age, and AED. A *p*-value below 0.05 was considered for statistical significance. The analyses were performed with IBM SPSS Statistics v22 and STATA v15.

## Results

A total of 157 consecutive patients (81% men), mean age 70 (range 50–87) years, were examined. AED of ≥10° was recorded in 291 joints (163 MCP and 128 PIP) and these were included in the analyses. The affected finger was the small (57%), ring (36%), middle (6%) and index (1%).

For all 291 joints mean AED was 46° (SD 21) and mean PED was 37° (SD 23). Examiner 1 measured 115 joints, examiner 2 measured 83 joints, and examiner 3 measured 93 joints (Table 1). Mean difference between AED and PED measured by examiner 1 was 6° (SD 6), by examiner 2 was 9° (SD 9), and by examiner 3 was 12° (SD 9). No statistically significant AED-PED differences were found according to joint (MCP mean 9 [SD 8], PIP 8 [SD 8]), finger (small 9 [SD 9], ring 9 [SD 7]), gender (men 10 [SD 10], women 9 [SD 7]), or age (*r* = − 0.04).

**Table 1:** Patient characteristics according to examiner

		Examiner 1	Examiner 2	Examiner 3
Number of patients		60	49	48
Number of joints		115	83	93
Men, n (%)		87 (76)	72 (87)	77 (83)
Women, n (%)		28 (24)	11 (13)	16 (17)
Patient age, mean (SD) yrs		69 (7.2)	70 (9.2)	70 (8.9)
Affected finger (n)	Index	0	0	2
	Middle	11	5	2
	Ring	34	37	35
	Little	70	41	54
MCP joint, ^a^ n		65	50	48
PIP joint,^a^ n		50	33	45
MCP joint, mean (SD)	AED	53 (24)	47 (16)	46 (22)
	PED	47 (16)	36 (21)	33 (25)
PIP joint, mean (SD)	AED	42 (21)	35 (19)	45 (19)
	PED	37 (21)	27 (22)	34 (20)

^a^Joints, in treated finger, with ≥10° AED at baseline

*AED* active extension deficit, *PED* passive extension deficit, *SD* standard deviation, *MCP* metacarpophalangeal, *PIP* proximal interphalangeal

The mixed effects model analysis showed that the identity of the examiner was a significant determinant of the AED-PED difference, with adjusted mean difference of 4.0, 6.5, and 2.5 for examiner 2 vs 1, examiner 3 vs 1, and examiner 3 vs 2, respectively (Table 2).

**Table 2:** Difference between active and passive extension deficit measured by 3 examiners

Examiner	Adjusted mean difference^a^	95% Confidence interval
2 vs 1	4.0	1.7–6.3
3 vs 1	6.5	4.2–8.8
3 vs 2	2.5	0.1–4.9

^a^Values are mean difference (degrees) between examiners in the difference between measured active and passive extension deficit, adjusted for affected joint, finger, patient age, patient gender, and active extension deficit (mixed effects model)

## Discussion

In patients with Dupuytren disease, ROM is the most common physical measurement used to classify disease severity and treatment efficacy [4]. The use of a goniometer to measure the joint extension deficit is regarded as objective and reliable [12]. In the literature, outcome measure in Dupuytren disease is reported as passive and/or active extension deficit; however a significant number of studies do not state how the extension deficit is measured [12]. This inconsistency in measurement methodology makes comparison between studies difficult [11].

In our study we measured both PED and AED in finger joint contractures caused by Dupuytren disease. The measurements were performed by three examiners (experienced hand therapists at the same orthopedic department). The AED-adjusted mean difference between AED and PED differed significantly among the three examiners. In most recent studies regarding treatment outcome in Dupuytren disease PED is used as the primary outcome measure [9, 10]. However, our study suggests that this measurement may be examiner-dependent and we believe that measurement of PED includes an additional examiner-related source of error compared to AED. This may be due to individual variations in the amount of pressure put on the digit when measuring passive extension. Although the measurements in this study were performed by three experienced hand therapists according to a standardized protocol, potential variation related to the effect of dynamism may not have been completely eliminated. Active extension may therefore be less examiner-dependent, and also may be a better illustrator of functional gain. Measuring active extension may also minimize the effect of dynamism [19].

Broekstra et al. [17] examined the inter-observer agreement between two examiners measuring total PED in joint contractures in patients with Dupuytren disease. The study reports a high inter-observer agreement, although the results varied according to examined finger, and before study start the two examiners evaluated 50 patients together to reach consensus in measurements. In our study, although the examiners discussed measurement technique they did not measure joints together before study start, which may explain the variability in measurements. This design may however increase the generalizability of our results, since we have evaluated the hand therapists in their daily clinical practice. A previous study by Engstrand et al. [16] in which several examiners measured joint ROM in the same patients with Dupuytren disease who had undergone surgery during the preceding year has shown that measurement of AED also varies among examiners, both in the MCP and PIP joints. Most previous studies that have examined reliability of finger joint ROM measurements were done on individuals without joint contracture caused by Dupuytren disease; we have not found other studies that have investigated reliability of joint ROM measurements in patients with Dupuytren disease prior to treatment.

One of the limitations of our study is the non-randomized allocation of patients to the examiners. However, the allocation was done without knowledge of the identity of the examining therapist that would be present at the particular treatment session. Moreover, no differences were found in any other patient characteristic. An alternative study design would have been to have all examiners measure the same patients in random order, in which case differences in AED and in PED between examiners could be analyzed. However, our results show that the difference between measured active and passive extension was similar in relation to all other variables (joint, finger, gender, and age) except for the identity of the examiner. Another limitation of our study is that all measurements were done on patients before treatment with collagenase injection. Measurements of contractures after treatment, or in patients undergoing surgical fasciectomy may differ, which may limit generalizability. However, at the study center collagenase injection was the first-line treatment for patients with Dupuytren joint contractures requiring treatment during the study period; few patients were treated with surgical fasciectomy whereas percutaneous needle fasciotomy is not used at the center.

## Conclusion

To our knowledge, our study is first to examine the difference between measured AED and PED in joints with Dupuytren disease. We have shown that the difference between measured AED and PED in joint contractures varies significantly between examiners. This highlights the need for standardization of measurements, including both active and passive extension deficit. Measurement consistency is important to enable comparison of results across studies evaluating treatment outcomes in patients with Dupuytren disease.

## Abbreviations

AED: Active extension deficit
MCP: Metacarpophalangeal
PED: Passive extension deficit
PIP: Proximal interphalangeal
ROM: Range of motion
